# Indoor radon monitoring through citizen science in Catalan schools: environmental assessment and educational outcomes

**DOI:** 10.3389/fpubh.2026.1953882

**Published:** 2026-10-26

**Authors:** Misbah Javed, Ian Martínez, Adrina Jimenez, Roser Sala Escarrabill, Arturo Vargas, Ferran Campillo i López, David Pino, Carlo Sabbarese, Claudia Grossi

**Affiliations:** 1 Department of Mathematics and Physics, University of Campania Luigi Vanvitelli, Caserta, Italy; 2 Institute of Energy Technologies (INTE), Universitat Politècnica de Catalunya (UPC), Barcelona, Spain; 3 Socio-Technical Research Centre, Centro de Investigaciones Energéticas, Medioambientales y Tecnológicas (CIEMAT), Universitat Autònoma de Barcelona, Barcelona, Spain; 4 Pediatric Environmental Health Specialty Unit (PEHSU), Olot and Garrotxa Regional Hospital Foundation, Olot, Spain; 5 Department of Physics, Universitat Politècnica de Catalunya (UPC), Barcelona, Spain; 6 Institute of Space Studies of Catalonia (IEEC-UPC), Castelldefels, Spain

**Keywords:** citizen science, environmental exposure, indoor radon, risk awareness, school environments

## Abstract

**Introduction:**

Indoor radon represents a significant environmental health hazard because high indoor radon concentration exposure increases lung cancer risk. Schools are critical settings due to prolonged occupancy by children. The RASCLET citizen-science project (Catalonia, Spain) integrated school-based radon monitoring with educational activities to evaluate exposure conditions and examine whether these activities increased radon knowledge and risk awareness. Meteorological variables and modeled radon flux were also explored in relation to indoor radon variability.

**Methods:**

Integrated radon measurements were conducted in 13 schools across Catalonia (Spain) during the winter of 2024–2025. Detectors were placed in classrooms on different floors. Schools represented different radon risk areas defined by official Spanish radon potential maps. At each school, approximately 40 students participated in educational activities about radon under their teacher’s supervision. Students and their teacher then selected the classrooms where detectors would be installed. Pre and post-intervention questionnaires assessed changes in students’ knowledge and perceptions of radon risks.

**Results:**

Indoor radon concentrations varied across schools and floor levels, with a median concentration of 49 Bq m^−3^ (interquartile range: 24–77 Bq m^−3^). All measurements obtained during the winter monitoring period remained below the 300 Bq m^−3^ reference level. PRE and POST-intervention questionnaires were completed by 538 and 339 students, respectively. The mean age was 11.07 ± 1.18 years (PRE) and 10.99 ± 1.04 years (POST), with female-to-male distributions of 49.4%/46.8 and 48.7%/48.7%, respectively. Correct identification of radon as a radioactive gas increased from 34.2 to 72.9% and recognition of its long-term health effects increased from 42.0 to 85.5% (both *p* < 0.001). Additionally, indoor radon data, combined with meteorological and radon flux data, were used in an exploratory modelling analysis. The analysis identified wind speed as the only statistically significant environmental variable associated with observed indoor radon concentrations (*p* = 0.025).

**Conclusion:**

These findings indicate that integrating school-based radon monitoring with citizen-science and educational activities can support exposure assessment, strengthen environmental health literacy, and improve radon risk communication in educational settings. This pilot study also provides a rationale for designing and conducting a larger-scale study across Spain to further evaluate this integrated approach.

## 1 Introduction

Natural radiation is the primary source of radiation exposure for the global population. Among these sources, radon (^222^Rn) is a major contributor and remains a primary concern in indoor environments (1). It originates from the decay of ^226^Ra in soils and rocks and can enter buildings through cracks and openings, where it may accumulate to levels that pose a significant health risk. Its short-lived decay products, combined with atmospheric aerosols, may reach lung tissues and, through alpha-particle emissions, damage lung cells. As a result, radon is recognized as the second leading cause of lung cancer after smoking (2–4). Worldwide, radon is estimated to account for 3–14% of lung cancer cases, with the relative risk increasing by approximately 16% for every 100 Bq m^−3^ increase in long-term indoor ^222^Rn concentration (5, 6). Based on these established health effects, regular monitoring of indoor radon is essential for public health protection.

Particular attention must be given to environments predominantly occupied by children, such as schools. Although lung cancer is primarily a disease of adulthood, children are generally considered more vulnerable to ionizing radiation due to their ongoing tissue development and their longer life expectancy, during which radiation-induced effects may manifest (7, 8).

To support effective monitoring and public health protection, many countries have adopted regulatory frameworks for radon control. In Europe, these requirements are defined in the Basic Safety Standards (BSS) Directive (9), which obliges Member States to establish reference levels for indoor radon and to identify radon priority areas where a significant proportion of buildings are expected to exceed those levels (10). In Spain, the BSS Directive has been transposed through Royal Decree 1029/2022, (11), which establishes a national reference level of 300 Bq m^−3^ (annual mean) for indoor radon in dwellings, buildings with public access, and workplaces. Within this framework, the Spanish Nuclear Safety Council (CSN) classifies municipalities according to radon potential based on official national cartography (12). The BSS Directive also requires Member States to establish national radon action plans addressing long-term risks from radon exposure, including communication strategies aimed at increasing public awareness and promoting appropriate preventive actions (9).

Beyond regulatory mapping, scientific studies have demonstrated substantial variability in indoor radon concentrations across Europe. Reported indoor radon levels range from a few tens of Bq m^−3^ to several thousand Bq m^−3^, depending on geological, structural, and environmental conditions, as well as occupants’ ventilation habits (13–15). Factors such as floor level, soil permeability, ventilation practices, and building materials significantly influence indoor radon accumulation. These findings highlight the importance of systematic monitoring in schools and workplaces to better understand local conditions and support effective prevention strategies.

Schools represent a priority setting for radon monitoring due to long occupancy times and the need to ensure preventive radiological protection for children. Moreover, monitoring activities conducted in educational environments can contribute to improving scientific literacy and environmental health awareness among students. However, while recent initiatives have strengthened citizen-science activities and radon communication strategies, further research is needed to better understand the specific impact of school-based monitoring initiatives on students’ awareness, engagement and protective behaviors.

Because formal monitoring programs cannot cover all buildings, complementary approaches such as citizen-science initiatives have been developed. In recent years, citizen-science methodologies have been increasingly applied in radon research to expand monitoring coverage and enhance public engagement (16, 17). In this context, citizen science extends beyond data collection by involving non-professional participants in scientific activities and facilitating knowledge exchange between researchers and communities. Previous radon initiatives have shown that participatory approaches can support radon testing while improving environmental health literacy, risk awareness, and communication among participants (16, 18). School-based initiatives may be particularly valuable because students and teachers can act as a bridge between scientific information, the school environment, and families.

European initiatives such as RadoNorm have promoted community participation in radon measurement campaigns (18). Previous citizen-science studies have involved citizens directly in radon testing and measurement activities, including the use of low-cost radon detectors (16, 17); however, these studies did not specifically integrate citizen participation with radon measurements analyzed within an ISO/IEC 17025-accredited laboratory framework. In contrast, the RASCLET project combines citizen-science activities with measurements analyzed within an ISO/IEC 17025-accredited laboratory, ensuring standardized measurement quality and reliability. Additionally, the specific contribution of school-based citizen-science initiatives to environmental health awareness remains insufficiently explored in Spain.

Furthermore, environmental conditions may also influence indoor radon variability. Meteorological factors such as outdoor temperature, atmospheric pressure, wind speed, and humidity affect ventilation patterns and pressure differentials between indoor and outdoor environments, thereby modifying radon entry from the soil and its indoor accumulation (19, 20). Seasonal variations have also been widely reported, with higher concentrations frequently observed during colder months (21, 22). Accounting for meteorological conditions is therefore important when interpreting temporal fluctuations in indoor radon concentrations.

Within this regulatory and scientific context, the RASCLET (Radon Awareness in Schools: Community Learning and Exposure Testing) project was developed by the Universitat Politècnica de Catalunya (UPC) in collaboration with the Centro de Investigaciones Energéticas, Medioambientales y Tecnológicas (CIEMAT) and the Pediatric Environmental Health Network in Catalonia (PEHSU. CAT). A pediatrician assigned to each participating school facilitated communication between the research team, teachers, and students throughout the project. The project combines professionally controlled radon measurements with the participation of the school community in educational and awareness activities. In parallel, environmental information, including available building-related characteristics, local meteorological data from the Catalan meteorological network, and modeled radon flux data from the European Radon Flux Maps, is used to contextualize the observed variability in indoor radon concentrations. The educational component further examines how participation is associated with students’ radon knowledge, risk awareness, and protective practices.

The aims of this study are: (1) to characterize integrated indoor radon levels in schools across different radon-potential zones in Catalonia (Spain) using integrated detectors analyzed by an ISO/IEC 17025-accredited laboratory; (2) to assess the educational impact of citizen participation on radon awareness and related health-protective behaviors; and (3) to examine the relationships between indoor radon variability, outdoor meteorological factors, and radon flux, while considering available qualitative information on classroom ventilation.

## 2 Methodology

### 2.1 Study area and participating schools

The study was conducted within the framework of the RASCLET project and involved predominantly primary schools, with approximately 33.3% of students in early secondary education, distributed across several municipalities in Catalonia (Spain). Schools were selected based on two predefined criteria: (i) a broad geographical distribution across the territory, and (ii) their classification within the official radon zoning map of the Spanish Nuclear Safety Council (CSN), which categorizes municipalities as either Zone 1 (lower radon potential) or Zone 2 (higher radon potential and priority action areas). The geographic coordinates (latitude and longitude) and corresponding CSN zone classification for each participating school are reported in Table 1. To preserve the confidentiality of the institutions, anonymized codes were assigned to indicate the different schools.

**Table 1:** Geographic coordinates (latitude and longitude) and corresponding CSN radon zoning classification (Zone 1 or Zone 2) of the participating schools included in the study.

School code	Latitude	Longitude	CSN zone
IEG	42.20	2.50	1
EPC	42.36	1.46	2
EPI	41.56	2.20	2
EIMF	41.72	2.93	2
IAR	42.81	0.75	2
EVP	41.85	3.13	2
EPEF	41.37	2.14	2
BUPU	41.36	2.10	1
ERO	41.44	2.17	2
EBE	41.39	2.16	2
EAI	41.40	2.17	1
ESSH	41.86	2.66	2
IDM	41.40	2.16	1

To further refine the environmental characterization of each site, local radon potential was assessed using percentile-based radon models (P90) obtained from the Spanish Urban Information System (SIU) (Supplementary Figure S1). These models provide fine-scale estimates of expected indoor radon concentrations based on geological and environmental determinants and complement the municipal-level CSN zoning (12). Radon potential values were assigned to each school based on its geographic location. This information was used to contextualize measured indoor radon concentrations and to support the interpretation of spatial variability among the schools.

### 2.2 Study design and workflow

The RASCLET project followed a structured workflow that combined indoor radon monitoring with a citizen-science educational approach. The study involved physicists responsible for radon measurements and data analysis, pediatric environmental health (PEH) professionals acting as health advisors and local trainers, and members of the educational community, including teachers and students. Pediatricians or pediatric nurses from the Pediatric Environmental Health Network in Catalonia (PEHSU. CAT) facilitated communication between the research team and participating schools and supported educational activities regarding radon and associated health risks.

Within RASCLET, students actively participated in the citizen-science component under the guidance of their teachers. Each participating classroom, typically comprising around 40 students, first carried out research on radon and its associated health effects under teacher guidance. Students and teachers then jointly discussed and selected the locations for detector installation within the school. Detector placement was subsequently carried out with the participation of students in small groups of approximately 6–8, following the measurement protocol provided by the research team. Pediatric environmental health professionals facilitated communication between the research team, schools, and families. Following the monitoring period, the measurement results were communicated to the participating schools and discussed with the school community during a closure day. Due to the young age of the students, radon measurement processing and scientific data analysis remained the responsibility of the research team.

The study phases included: (1) initial coordination and training between the research team, teachers, and pediatricians, (2) administration of the pre-intervention questionnaire to the students, (3) educational activities on radon and its associated health effects conducted by the students under teacher guidance, (4) selection of detector locations and implementation of indoor radon monitoring in schools, (5) a three-month radon monitoring period, (6) administration of the post-intervention questionnaire to the students, (7) data analysis, and (8) dissemination of results to participating schools and the wider community.

Initial coordination included an online introductory webinar in which the study objectives, measurement protocol, and educational components were presented to participating schools and health professionals. Teachers were provided with a detailed protocol for detector deployment, together with a table to record the required information for each measurement location. Following the students’ research activities on radon and its health effects, students and teachers jointly selected the detector locations and carried out the placement following the research-team protocol. Pediatric environmental health professionals supported communication with families and school staff. At the end of the monitoring period, a second webinar was organized to present the results and facilitate discussion and questions among all participants.

Environmental measurements and educational activities were conducted in parallel throughout the study. The chronological distribution of the study activities across the 2024–2025 academic year is summarized in Supplementary Table S1, illustrating the temporal sequence and overlap of the different phases.

### 2.3 Indoor radon measurement protocol

#### 2.3.1 Detectors description

Indoor radon concentrations were measured using Radtrak^3^® passive alpha-track detectors supplied and analyzed by Radonova Laboratories, Sweden (23), an ISO 17025 accredited laboratory. These detectors are based on solid-state nuclear track detection, in which alpha particles emitted during the decay of radon and its short-lived progeny create latent tracks on a polymer film housed within the detector. After the exposure period, the film is chemically etched, and the tracks are counted in the laboratory, providing a time-integrated average radon concentration and its uncertainty (24).

#### 2.3.2 Indoor detector deployment

Each participating school received five Radtrak^3^ detectors. Four detectors were installed in classrooms or other regularly occupied indoor spaces, while one detector served as a transport control (background) detector. The background detector remained sealed during the exposure period and was opened only after being returned to the laboratory. It was used according to the study protocol to account for background track formation not attributable to indoor radon exposure. Room selection followed predefined criteria to ensure representative sampling of occupant exposure, including regular daily occupancy, general ventilation conditions and floor level. Detectors were positioned in accordance with standard radon-measurement guidelines (25): at least 0.5–1 m above the floor, away from windows, doors, radiators or other heat sources, avoiding direct airflow and placed on a stable horizontal surface. The detectors were deployed for approximately 90 days during the winter monitoring campaign (January–March 2025), consistent with recommendations for measurements over a period of at least 3 months to better characterize long-term indoor radon exposure (2). The reported indoor radon concentrations represent the time-integrated mean concentrations measured during this exposure period; no seasonal correction factors were applied to estimate annual mean concentrations.

#### 2.3.3 Deployment metadata collection

Metadata were recorded for each detector, including detector identification, school and municipality, GPS coordinates, floor level, room information, reported ventilation conditions, and average daily occupancy. These data were collected to document the measurement context and support the interpretation of indoor radon variability. A summary of the recorded metadata is provided in Supplementary Table S2.

#### 2.3.4 Detector handling and laboratory analysis

At the end of the exposure period, all Radtrak^3^ detectors were sealed and prepared for laboratory processing. In accordance with the study protocol, the background (transport-control) detector was analyzed together with the exposed detectors and used for background correction of the measured radon concentrations. The detectors were analyzed following the standardized ISO 11665-4 methodology for long-term passive radon measurements. The procedure included chemical development of the track-etch film, automated track counting and the application of the batch-specific calibration factors (26). The final indoor radon concentrations were reported in units of Bq m^−3^ and provided with quality-control information for subsequent statistical analysis. Exposure times were verified for consistency and detectors with incomplete metadata or improper deployment conditions were excluded. Standard placement rules were applied uniformly across all schools to minimize variability.

### 2.4 Meteorological parameters

Outdoor meteorological parameters were included to characterize the environmental conditions potentially influencing indoor radon accumulation and transport (27–31). Meteorological data were obtained from the Automatic Weather Station Network (XEMA) of the Catalan Meteorological Service, which provides high-quality, continuous measurements with extensive spatial coverage across Catalonia (32).

For each participating school, a representative meteorological station was assigned based on geographical proximity, data completeness and similarity in local environmental conditions. In cases where the closest station had incomplete or discontinuous data records, an alternative station with more reliable data was selected to ensure temporal continuity and representativeness of the meteorological conditions at the school location.

The meteorological variables considered in this study were surface atmospheric pressure, 2-m air temperature, 2-m relative humidity and 10-m wind speed. Meteorological data were recorded at a 30 min temporal resolution and aggregated to daily mean values. The daily meteorological values were subsequently averaged over the approximately three-month radon monitoring period to obtain a representative mean value for each school, allowing direct comparison with the corresponding time-integrated indoor radon concentration. For consistency with the indoor radon measurements, analyses were restricted to the period corresponding to the radon monitoring campaign (January–March 2025). The meteorological stations selected for each school, along with their distances, are summarized in Table 2.

**Table 2:** List of participating schools associated with meteorological stations from the XEMA network, and distances between each school and its corresponding station.

School abbreviation	Assigned meteorological station code	Distance school–station (m)
IEG	YB	2.473
EPC	CD	2.577
EPI	XG	14.789
EIMF	DO	12.870
IAR	CU	727
	YN	254
EVP	DO	9.676
EPEF	X9	2.884
BUPU	X8	1.923
ERO	VQ	12.420
EBE	UP	3.222
EAI	YA	646
ESSH	XS	432
IDM	X4	3.949

### 2.5 Radon flux

Soil radon exhalation was included as an environmental parameter to characterize the potential source of radon entering buildings from the ground. Radon flux data were obtained from the European daily radon flux dataset developed within the European traceRadon project (33), which utilized the diffusion model described by Karstens et al. (34). The model integrates a dataset of geological and environmental parameters, including soil uranium content, soil properties, and soil moisture derived from ERA5-Land reanalysis data to estimate daily soil radon exhalation rates across Europe.

The dataset has a spatial resolution of 0.05° × 0.05° in latitude and longitude. Flux values were assigned to each participating school based on their geographic coordinates by extracting the corresponding grid-cell values from the daily radon flux maps and were averaged over the indoor radon monitoring period to obtain a representative mean value for each school over the measurement months. Figure 1 illustrates the spatial distribution of radon flux across Catalonia and the locations of the participating schools (black circles) and the meteorological stations (red triangles), providing a geographical overview of soil radon exhalation patterns within the study area.

**Figure 1:**
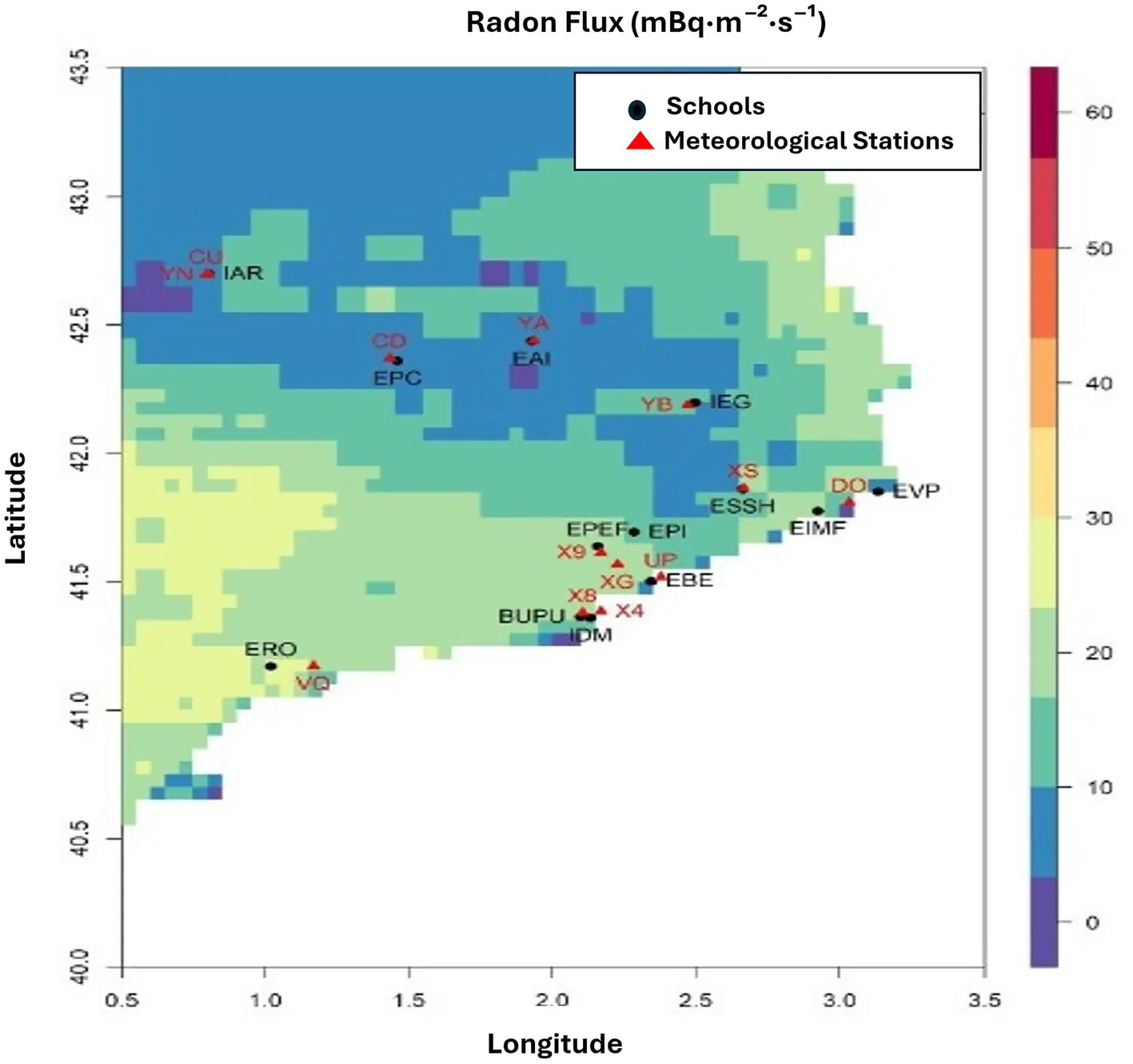
Spatial distribution of soil radon flux across Catalonia derived from the European daily radon flux dataset. Red triangles indicate the locations of the meteorological stations and black circles show the schools positions.

### 2.6 Radon potential

Radon potential, derived from the CSN map, was included to provide a geological and spatial context for the interpretation of indoor radon concentrations measured in the participating schools. Radon potential classification was derived from the official cartography developed by the Spanish Nuclear Safety Council (12), which classifies the national territory according to the 90th percentile (P90) of indoor radon concentrations measured in buildings, with particular emphasis on ground and first-floor levels. This percentile-based indicator represents the radon concentration exceeded by only 10% of buildings within a given area and is used to identify zones with higher expected radon exposure (35, 36).

Each participating school was geolocated using the CSN radon potential map, and the corresponding P90 category and radon zoning class (Zone 1 or Zone 2) were assigned based on geographic position.

In addition to the CSN classification, radon exhalation rates were obtained from the traceRadon daily radon flux dataset for 2022, the most recent validated dataset available at the time of analysis (33). The exhalation rate (mBq m^−2^ s^−1^) represents the modeled soil radon flux and provides a continuous quantitative indicator of radon source strength across the territory. The radon potential categories (P90) and corresponding exhalation rates assigned to each participating school are summarized in Table 3.

**Table 3:** Radon potential classification (P90), and modeled radon exhalation rates (mBq m^−2^ s^−1^) derived from the trace radon daily radon flux dataset for 2022 (33) for each participating school.

School code	Radon potential (P90) (Bq m^−3^)	Exhalation rate (mBq m^−2^ s^−1^)
IEG	101–200	9.3
EPC	201–300	4.3
EPI	201–300	10.4
EIMF	>400	14.0
IAR	201–300	6.7
EVP	>400	4.9
EPEF	>400	18.1
BUPU	201–300	20.0
ERO	201–300	20.3
EBE	>400	15.8
EAI	201–300	4.8
ESSH	201–300	16.6
IDM	201–300	20.0

### 2.7 Online surveys for educational impact

To assess changes in students’ knowledge, perceptions, and behaviors related to radon, structured online questionnaires were administered before and after the educational and measurement campaign. All participating classes completed the pre-intervention (PRE) questionnaire prior to the radon-related activities. However, due to attrition, not all students participated in the post-intervention (POST) survey. Consequently, a total of 538 students completed the PRE questionnaire, while 339 students completed the POST questionnaire.

Data were analyzed at the question level using aggregated responses, and individual students were not matched between the PRE and POST surveys. Accordingly, the PRE and POST questionnaires were treated as independent samples for statistical comparison. The POST questionnaire was administered after completion of the radon-related educational activities conducted within the participating classes. The questionnaires evaluated demographic information, factual knowledge, self-perceived understanding, risk perception, awareness of protective actions, and radon-related behaviors. Surveys were administered through anonymous Google Forms, and all responses were collected anonymously. The structure of the questionnaires is summarized in Supplementary Figure S2.

### 2.8 Statistical analysis

Statistical analyses were conducted to summarize indoor radon concentrations and explore their associations with environmental variables and survey data collected within the RASCLET study. Integrated indoor radon concentrations were described using summary statistics (mean, median, standard deviation and interquartile range) and graphical methods to assess variability across schools and floor levels (37). For analyses of the relationships between indoor radon and environmental variables, the individual detector measurements were summarized at the school level so that each school contributed one representative indoor radon value. This was necessary because meteorological parameters and radon flux were assigned at the school or local level. Consequently, the school, rather than the individual detector, was considered the independent unit of analysis (*n* = 13). Associations between school-level indoor radon concentrations and environmental parameters were explored using correlation and regression analyses. Given the limited number of independent school-level observations, these analyses were considered exploratory and were not intended for the development or validation of a predictive model. Because the passive detectors provided radon concentrations integrated over the approximately 90-day monitoring period, the environmental analyses were limited to between-school associations between integrated indoor radon concentrations and environmental conditions summarized over the corresponding monitoring period. They were not intended to assess short-term or temporal meteorological effects on indoor radon dynamics.

Questionnaire responses were analyzed as independent PRE and POST samples because individual participants could not be matched between surveys. Inferential PRE–POST comparisons were restricted to questionnaire items with directly comparable wording and constructs in both surveys. Binary items were compared using Pearson’s chi-square test, whereas comparable Likert-scale items were evaluated using the Mann–Whitney U test. Items that differed in wording or construct between the PRE and POST questionnaires were reported descriptively and were not subjected to direct inferential comparison. Cramér’s V was used as an effect-size measure for chi-square comparisons, while rank-biserial correlation was used for Mann–Whitney U comparisons. Statistical significance was defined as *p* < 0.05.

Data cleaning and preliminary descriptive analyses were performed using Microsoft Excel. All statistical analyses and modeling were conducted using the R statistical environment (RStudio).

## 3 Results

### 3.1 Indoor radon concentrations

This section presents the results of indoor radon measurements conducted in the participating schools. Indoor radon concentrations are first summarized at the school level and subsequently examined in relation to radon zoning and variability associated with building characteristics, particularly floor level and ventilation.

#### 3.1.1 Indoor radon measurements in schools

Indoor radon concentrations were measured in the 13 schools participating in the study. In total, 52 passive detectors were deployed (four per school) in regularly occupied rooms. Five detectors failed to provide a valid reading of concentration values and were consequently excluded from the analysis. Measurements reported below the limit of detection (LOD, 10 Bq m^−3^) were assigned a substitution value equal to half the LOD (5 Bq m^−3^) for statistical analysis. As a result, data from only 47 detectors were included in the final evaluation.

Indoor radon concentrations were summarized at the school level, indicating the number of valid detectors, mean radon concentration, and standard deviation (Table 4). Schools were classified according to their CSN radon zoning category (Zone 1 or Zone 2) to provide the necessary geological context for the observed variability.

**Table 4:** School-level summary of indoor radon concentrations, including CSN radon zone, number of valid detectors, mean radon concentration, and standard deviation.

School code	CSN zone	Valid detectors (n)	Mean (Bq m^−3^)	SD
IEG	1	3	163	47
EPC	2	4	95	55
EPI	2	4	109	69
EIMF	2	4	42	10
IAR	2	3	30	11
EVP	2	4	31	12
EPEF	2	4	74	39
BUPU	1	4	21	5
ERO	2	4	74	47
EBE	2	4	54	21
EAI	1	4	42	15
ESSH	2	3	66	19
IDM	1	2	22	4

Mean indoor radon concentrations varied considerably across schools, with school-level means ranging from 21 Bq m^−3^ (BUPU, Zone 1, exhalation rate: 20 mBq m^−2^ s^−1^) to 163 Bq m^−3^ (IEG, Zone 1, exhalation rate: 9 mBq m^−2^ s^−1^). Notably, the highest school mean was observed at IEG (Zone 1), while several schools in Zone 2 exhibited moderate-to-low means (e.g., EVP, IAR, and EIMF).

When compared with the modeled radon exhalation rates derived from the 2022 dataset (Table 3), the results indicate that soil exhalation alone does not directly account for the variability observed in indoor concentrations. For instance, BUPU and IDM exhibit some of the highest exhalation rates (both 20 mBq m^−2^ s^−1^) but relatively low indoor concentrations (21 and 22 Bq m^−3^), whereas IEG shows the highest indoor radon concentration despite a moderate exhalation rate.

Based on the mean values reported in Table 4, most schools exhibited concentrations below the WHO reference level of 100 Bq m^−3^. However, two schools (IEG and EPI) exceeded this threshold, with IEG showing the highest mean value of approximately 163 Bq m^−3^. All school-level mean concentrations were below the Spanish reference level of 300 Bq m^−3^. The approximately 90-day monitoring campaign was conducted from January to March 2025, within the October–May period established by the Spanish regulatory framework for estimating annual average radon concentrations from measurements of at least 3 months (38). Accordingly, the measured concentrations were used without applying an additional seasonal correction factor.

To complement the school-level summary values, boxplots were generated to illustrate the variability of indoor radon concentrations within each school (Figure 2). This visualization allows for the evaluation of the distribution of measurements, including median values, interquartile ranges, and potential outliers.

**Figure 2:**
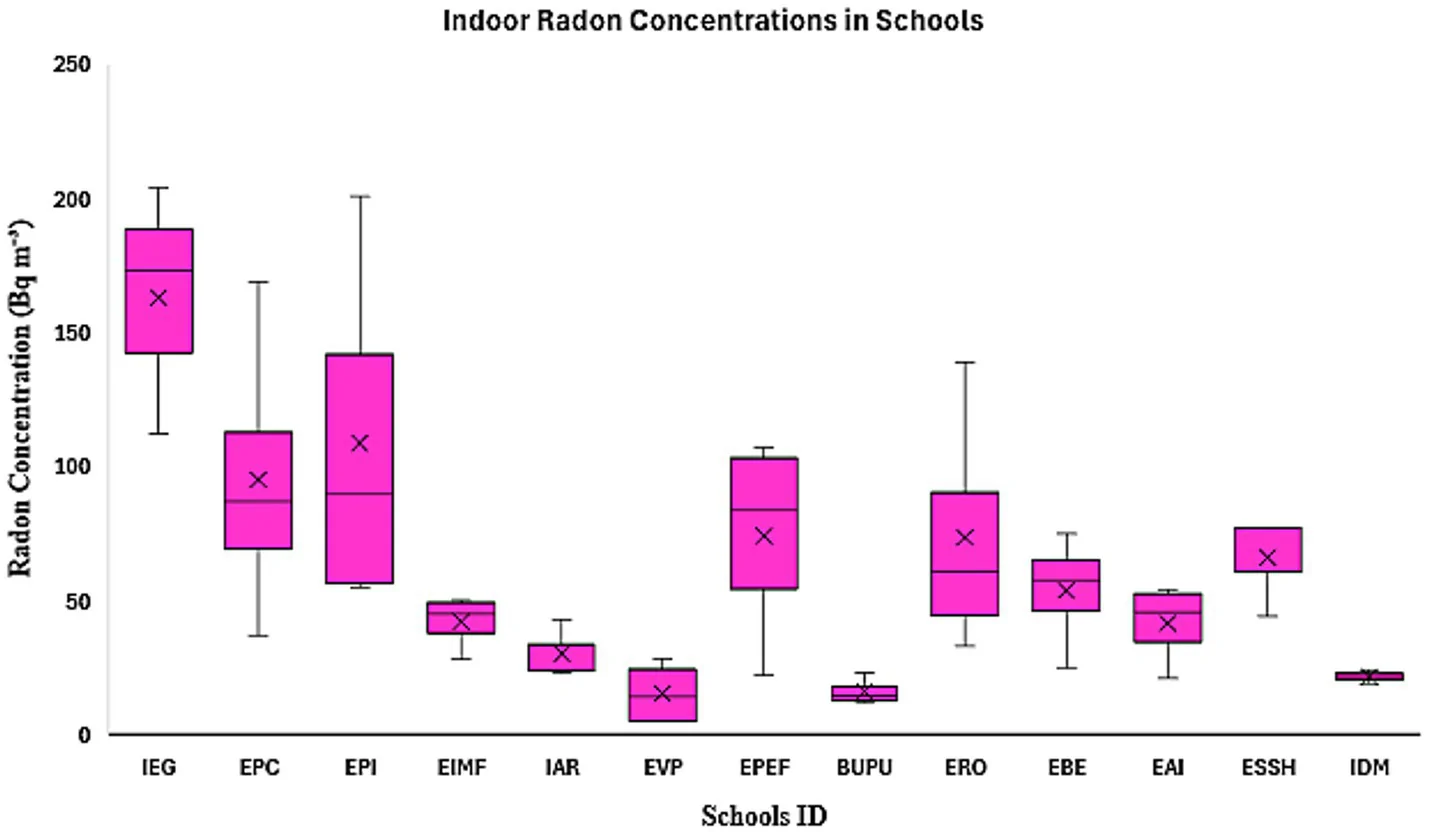
Boxplot summary of indoor radon concentrations measured in participating schools. Boxes represent the interquartile range, horizontal lines indicate median values, whiskers denote the minimum and maximum concentrations measured, and crosses indicate mean values.

The boxplots reveal that the spread of indoor radon concentrations varies substantially among schools. Some schools exhibit relatively compact distributions, whereas others show wider interquartile ranges and higher upper values, indicating greater dispersion of measured concentrations within those institutions.

#### 3.1.2 Distribution pattern of measured values

To examine the overall distribution of measured indoor radon concentrations across all schools, a frequency histogram was constructed (Supplementary Figure S3). The histogram shows that most measurements fall below 100 Bq m^−3^, with the highest frequency occurring in the lowest concentration interval (0–50 Bq m^−3^). Only a small number of observations exceed 100 Bq m^−3^, and very few values are above 150 Bq m^−3^. The distribution is positively skewed, indicating that while most concentrations are relatively low, a limited number of higher measurements form a right-tailed distribution.

#### 3.1.3 Influence of floor level on indoor radon concentration

Radon concentrations were examined according to floor level to characterize how measured indoor radon values vary with elevation within school buildings (Figure 3). Of the 47 valid measurements, 11 were obtained from basement rooms, 27 from ground-floor rooms, 6 from first-floor rooms, 2 from second-floor rooms, and 1 from a third-floor room.

**Figure 3:**
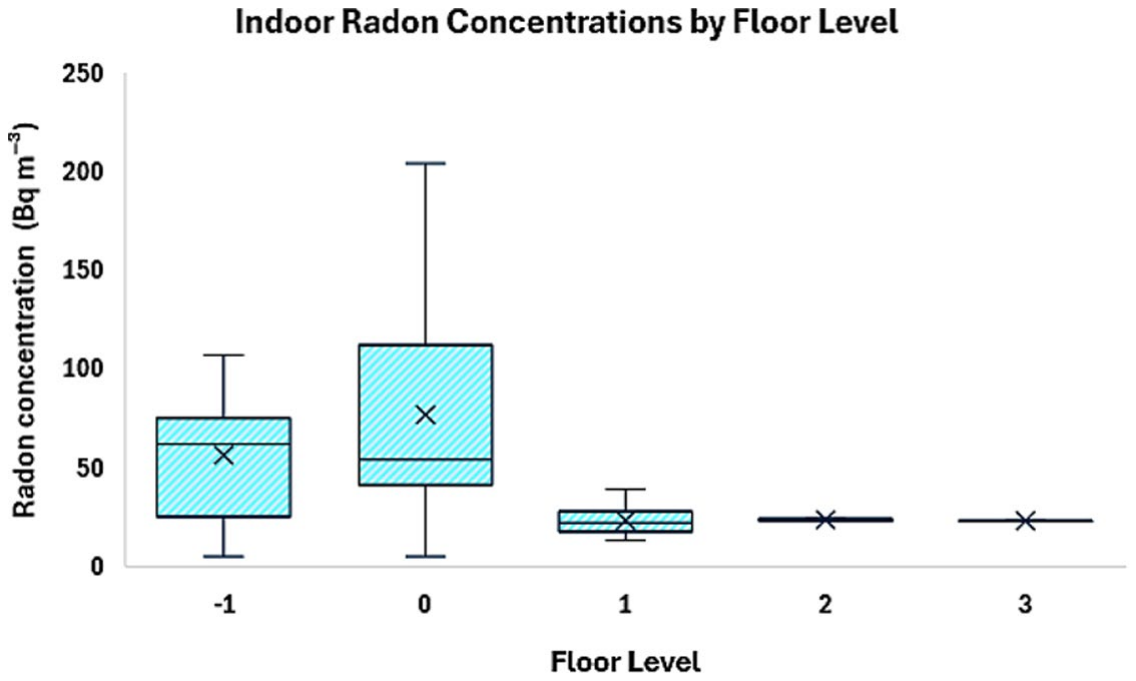
Indoor radon concentrations measured at different floor levels. Boxes represent the interquartile range, horizontal lines indicate median values, whiskers denote minimum and maximum values, and crosses indicate mean concentrations. [Number of measurements per floor level: basement (*n* = 11), ground floor (*n* = 27), first floor (*n* = 6), second floor (*n* = 2), and third floor (*n* = 1)].

The distribution of indoor radon concentrations indicates a tendency toward higher concentrations at lower building levels. Basement (−1) and ground-floor (0) rooms show both higher concentrations and greater dispersion compared with measurements obtained on upper floors (≥1). The ground floor exhibits the widest interquartile range, suggesting substantial variability in radon concentrations at this level. In contrast, measurements obtained on higher floors generally showed lower concentrations, although the number of observations was limited, particularly for the second and third floors. Therefore, while the available measurements suggest lower concentrations at higher floors, this pattern should be interpreted cautiously due to the limited number of measurements at these elevations.

Because radon entry from the soil is expected to have a stronger influence on rooms in direct or close contact with the ground, basement and ground-floor measurements are particularly relevant when interpreting associations with radon potential and modeled radon flux. In the present dataset, 38 of the 47 valid measurements were obtained from basement or ground-floor rooms. Given the limited number of participating schools, floor-specific inferential analyses were not performed, and the influence of floor level was instead examined descriptively.

Information on classroom ventilation was collected qualitatively; however, the information obtained was not sufficiently detailed for inclusion in the statistical analysis. In future studies, more detailed information on ventilation conditions will be requested from participants.

### 3.2 Environmental parameters and exploratory analysis

#### 3.2.1 Environmental parameters during the monitoring period

To contextualize the integrated indoor radon measurements and support the subsequent analysis of potential environmental influences, the primary meteorological parameters characterizing the monitoring period were examined. Temperature, relative humidity, atmospheric pressure, and wind speed were obtained from the meteorological stations corresponding to each school location and summarized as mean values over the measurement period (Table 5). These parameters represent external environmental conditions that may influence indoor radon dynamics through air exchange processes (including natural ventilation), pressure-driven soil-gas transport, and atmospheric stability.

**Table 5:** Mean values of meteorological parameters (temperature, relative humidity, atmospheric pressure, and wind speed) recorded at the meteorological stations associated with each school during the monitoring period (January–March 2025).

School	Weather station code	Temperature (°C)	Relative humidity (%)	Atmospheric pressure (hPa)	Wind speed (m s^−1^)
IEG	YB	7.82	75.63	966.11	1.35
EPC	CD	5.26	72.97	919.34	1.16
EPI	XG	10.28	77.95	1003.93	1.80
EIMF	DO	10.51	79.73	1016.45	2.62
IAR	CU	4.94	70.88	899.55	1.33
EVP	DO	10.51	79.73	1016.45	2.62
EPEF	X9	9.16	79.98	996.60	1.74
BUPU	X8	11.90	71.60	1008.20	2.14
ERO	VQ	10.59	74.55	1004.30	2.24
EBE	UP	11.17	76.60	1008.04	2.14
EAI	YA	3.91	67.53	879.21	1.44
ESSH	XS	9.56	77.44	Not available	1.87
IDM	X4	12.80	66.73	1013.91	2.02

Table 5 reports the mean values of the selected meteorological variables for each school during the period from January to March 2025. Overall, the observed values are consistent with typical winter–early spring climatic conditions in the study area, characterized by moderate temperatures, relatively high relative humidity, and generally stable atmospheric pressure.

Atmospheric pressure data were not available for the XS meteorological station associated with ESSH; therefore, this school was excluded from analyses requiring atmospheric pressure. Other observations with missing radon or environmental data were similarly excluded from the corresponding exploratory analyses. These missing data did not affect the descriptive characterization of environmental conditions.

#### 3.2.2 Temporal behavior of meteorological parameters: example of station YA

To illustrate the temporal variability of the environmental parameters, Supplementary Figure S4 presents, as an example, the time series of temperature, relative humidity, atmospheric pressure, and wind speed recorded at a representative meteorological station (YA) from 2018 to 2025. This station serves as an example to demonstrate the typical seasonal patterns and short-term variability characterizing the study area.

The combined time series in Supplementary Figure S4 highlights a clear annual cycle in air temperature, with minimum values in winter and maximum values in summer, reflecting the expected Mediterranean climatic regime. Transitional seasons are characterized by intermediate temperatures and enhanced variability. Relative humidity exhibits pronounced short-term fluctuations throughout the year, with generally higher values during winter and greater variability during warmer periods. Atmospheric pressure remains relatively stable on a seasonal scale, while exhibiting episodic short-term drops associated with synoptic meteorological events. Wind speed displays strong short-term variability, with intermittent high-velocity episodes superimposed on lower background values and a minimal seasonal dependence.

Overall, the temporal patterns shown in Supplementary Figure S4 provide broader seasonal context for the meteorological conditions during the indoor radon monitoring period.

#### 3.2.3 Correlation analysis between radon concentration and environmental parameters

While the descriptive analysis provides essential contextual information, it does not allow for a quantitative assessment of the relationships between indoor radon concentrations and environmental conditions. Therefore, a correlation analysis was performed to evaluate the strength and direction of linear associations between radon concentrations and the primary meteorological variables: temperature, relative humidity, atmospheric pressure, wind speed, and radon flux derived from the European radon flux maps (Figure 4).

**Figure 4:**
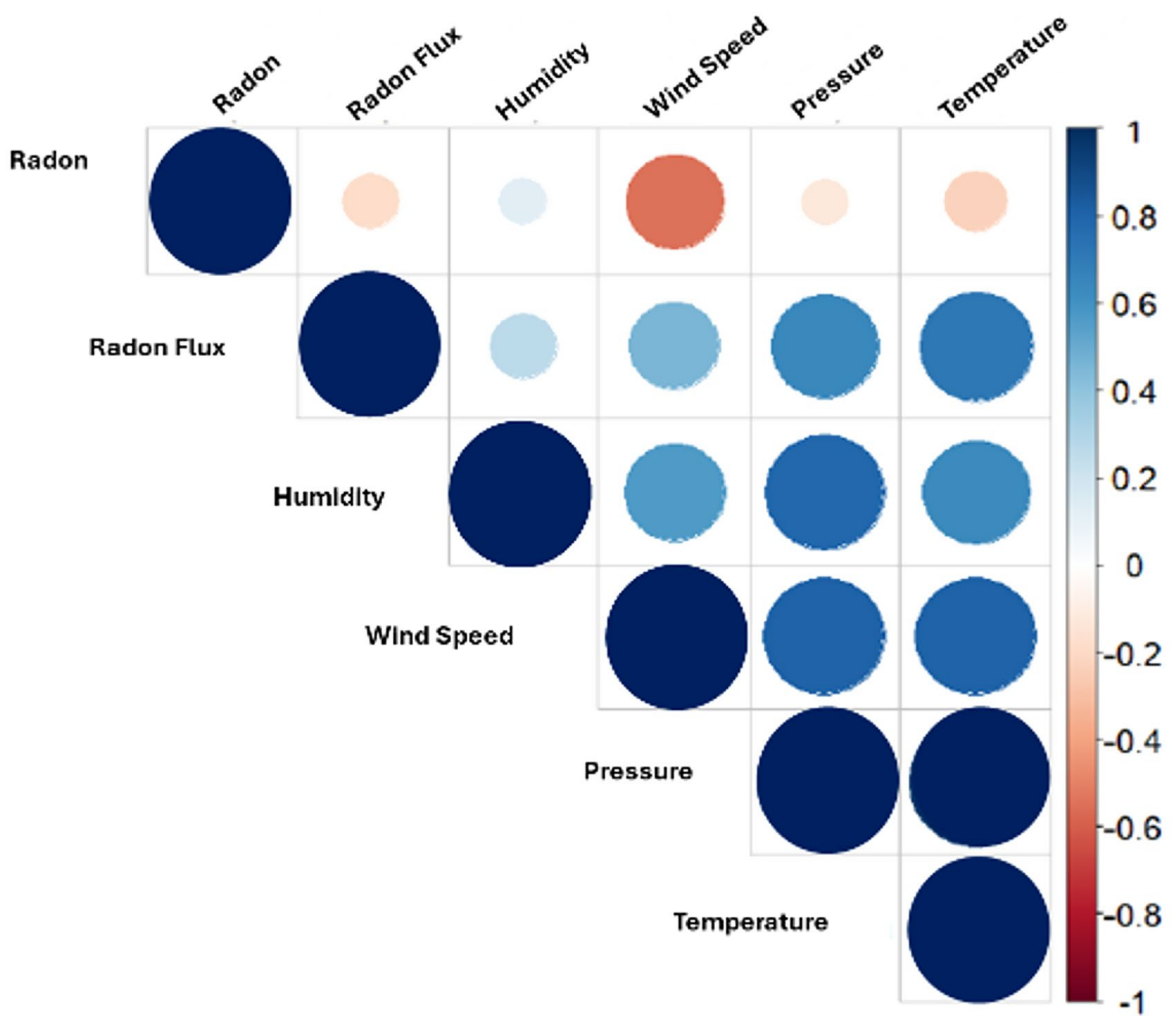
Correlation matrix between indoor radon concentration, radon flux, and environmental parameters (relative humidity, wind speed, atmospheric pressure, and temperature). Circle size and color indicate the strength and direction of correlation coefficients, with blue indicating positive correlations and red indicating negative correlations.

The correlation matrix shown in Figure 4 indicates generally weak-to-moderate linear correlations between indoor radon concentrations and individual environmental parameters. In contrast, strong and statistically significant correlations are observed among several meteorological predictors themselves, particularly between temperature, atmospheric pressure, relative humidity, and wind speed. These interdependencies indicate multicollinearity among the environmental variables, which can complicate the interpretation of simple bivariate relationships. Given the limited number of independent school-level observations, these associations were interpreted as exploratory.

#### 3.2.4 Exploratory multivariable analysis

Multivariable analyses were performed as a secondary exploratory assessment of the relationships between environmental parameters and school-level indoor radon concentrations. A multiple linear regression including relative humidity, atmospheric pressure, air temperature, wind speed, and radon flux showed that wind speed was the only variable significantly associated with indoor radon concentration (*β* = −102.022, *p* = 0.025), whereas the other predictors were not statistically significant (Table 6). Variance Inflation Factors indicated substantial multicollinearity for relative humidity, atmospheric pressure, and air temperature, while wind speed and radon flux showed comparatively lower values (Supplementary Table S3).

**Table 6:** Regression coefficients (*β*) and associated *p*-values for the full multiple linear regression model relating indoor radon concentration to environmental predictors.

Predictor	Coefficient (*β*)	*p*-value (Pr (>|t|))
Intercept	−2.92 × 10^3^	0.124
Humidity relative	−1.07 × 10^1^	0.323
Pressure	4.63 × 10^0^	0.144
Temperature	−5.76 × 10^1^	0.194
Wind speed	−1.02 × 10^2^	**0.025**
Radon flux	−1.39 × 10^0^	0.588

Ridge regression was additionally applied to reduce coefficient instability associated with multicollinearity, using atmospheric pressure, air temperature, and wind speed based on the preceding analyses. Measured and Ridge-estimated indoor radon concentrations are presented in Table 7; estimates were unavailable for ESSH and IDM due to incomplete environmental predictor data. The Ridge estimates broadly reflected differences in indoor radon concentrations among schools, although discrepancies were observed for some locations. Given the small number of independent schools (*n* = 13), these multivariable analyses were considered exploratory and were not intended for prediction or model validation.

**Table 7:** Comparison between measured indoor radon concentrations (IRC) and Ridge-estimated values for the analyzed schools.

School	Measured IRC [Bq m^−3^]	Ridge-estimated IRC [Bq m^−3^]	Error (%)
IEG	163	107	34
EPC	95	99	4
EPI	109	91	17
EIMF	42	32	24
IAR	30	71	137
EVP	31	32	3
EPEF	74	93	26
BUPU	21	57	171
ERO	74	52	30
EBE	54	60	12
EAI	42	52	24
ESSH	66	N/A	N/A
IDM	22	N/A	N/A

### 3.3 Questionnaire results and educational impact

#### 3.3.1 Participant overview

A total of 538 students completed the pre-intervention (PRE) questionnaire, while 339 students participated in the post-intervention (POST) survey. The reduction in sample size between the PRE and POST assessments was primarily due to logistical constraints, including participant availability for the post-intervention survey during the summer season.

The students’ ages spanned primary and secondary school levels, enabling subsequent age-stratified analyses of learning outcomes, risk perception, and satisfaction with the educational intervention. Age, school affiliation and gender were the demographic variables considered, providing the basis for the comparative analyses of knowledge accuracy, awareness, and behavioral outcomes, presented in the following sections.

#### 3.3.2 Knowledge accuracy (true/false questions)

Students’ factual knowledge regarding radon was assessed using seven True/False questions. Responses were coded as correct or incorrect, with “I don’t know” answers classified as wrong. For each question, the percentage of correct responses was calculated and compared between the pre-intervention (PRE) and post-intervention (POST) surveys to evaluate the gain in knowledge following the educational activity (Figure 5).

**Figure 5:**
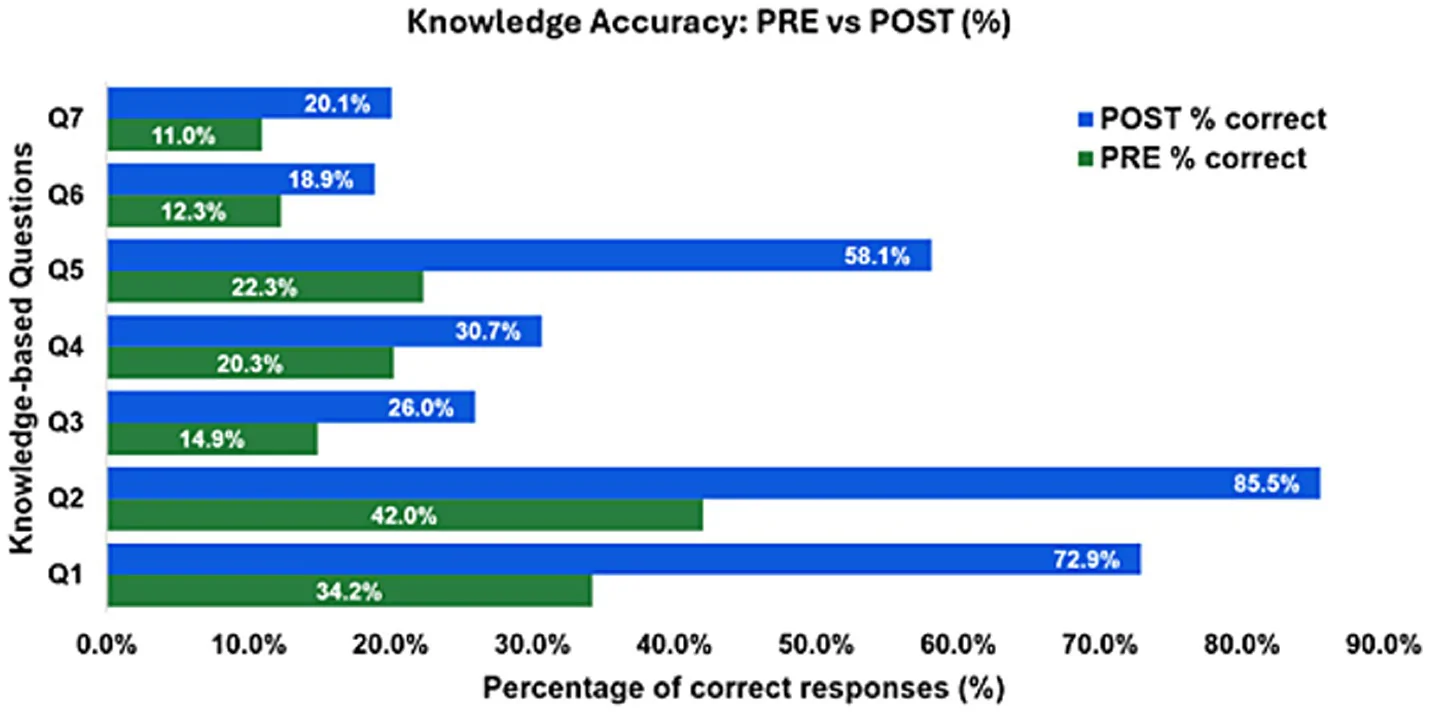
Knowledge accuracy before and after the educational intervention.

Figure 5 illustrates the proportion of correct answers for each knowledge-based question before and after the classroom-based intervention and the teacher training activities within the classrooms. Overall, a clear improvement in knowledge accuracy was observed across all questions in the POST survey compared with the PRE survey. The most pronounced increases corresponded to questions addressing fundamental concepts of radon. In particular, the proportion of students correctly identifying radon as a radioactive gas (Q1) increased from 34% in the PRE survey to 73% in the POST survey. Similarly, correct responses to the question concerning the long-term health effects of radon exposure (Q2) rose substantially, from 42% before the intervention to 86% afterward.

Moderate but consistent improvements were also observed for questions related to radon properties and dynamics. Correct identification of radon as a non-flammable gas (Q3) increased from 15 to 26%, while awareness that radon concentrations can be reduced (Q5) showed a marked increase from 22 to 58%. Smaller yet notable gains were recorded for questions addressing radon accumulation in buildings (Q4) and misconceptions related to radon origins and sensory characteristics (Q6 and Q7), indicating a partial correction of common misunderstandings. Item-level chi-square analyses indicated statistically significant PRE–POST differences for all seven knowledge items (all *p* < 0.05), with the largest changes observed for the core concepts of radon radioactivity, long-term health effects, and the possibility of reducing indoor radon concentrations. Detailed test statistics, *p*-values, and effect-size estimates are reported in Supplementary Table S4.

#### 3.3.3 Awareness of radon health risks, protective actions, and related behaviors

Students’ awareness of radon-related health risks and protective actions was evaluated using Yes/No questions administered before (PRE) and after (POST) the educational intervention. Two complementary dimensions were analyzed:

- Awareness of radon-associated health risks and the existence of protective actions.
- Behavioral and communication outcomes related to radon.

Changes in awareness and behavior were assessed by comparing the proportions of affirmative (“Yes”) responses between the PRE and POST surveys (Figures 6A,B).

**Figure 6:**
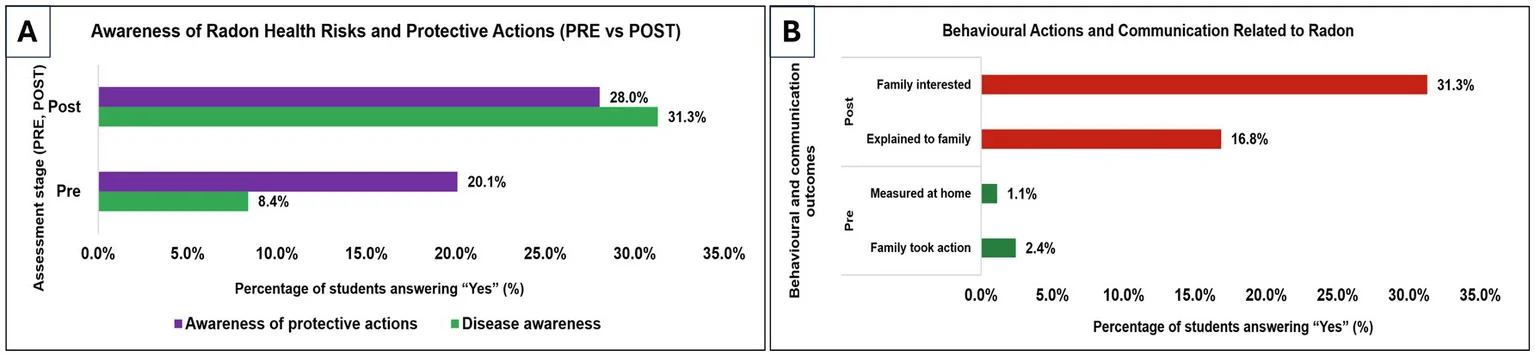
Awareness, behavioral, and communication outcomes related to radon before (PRE) and after (POST) the educational intervention: **(A)** awareness of radon-associated health risks and protective actions; **(B)** behavioral and communication outcomes, including family communication, interest, and action regarding radon measurement.

As shown in Figure 6A, awareness of radon-related health risks and protective actions increased markedly following the intervention. In the PRE survey, awareness levels were low, with only 8.4% of students reporting knowledge of radon-associated diseases and 20.1% indicating awareness of potential protective actions. After the intervention, these values increased to 31.3 and 28.0%, respectively, reflecting an improvement in baseline awareness. For these two directly comparable binary items, Pearson’s chi-square tests confirmed a significant increase in awareness of radon-associated diseases (8.4% vs. 31.3%, *p* < 0.001) and a smaller but statistically significant increase in awareness of protective actions (20.1% vs. 28.0%, *p* = 0.007). Detailed test statistics and effect-size estimates are reported in Supplementary Table S4. Behavioral and communication outcomes are illustrated in Figure 6B. Prior to the intervention, only a small proportion of students reported concrete actions, such as measuring radon at home (1.1%) or their families taking protective measures (2.4%). In contrast, the POST survey revealed increased engagement at the household level, with 17% of students reporting that they had explained radon-related issues to their families, and 31% indicating family interest in radon measurement. Because the PRE and POST behavioral questions differed in wording and measured different constructs, these outcomes are presented descriptively and were not subjected to direct PRE–POST statistical comparison.

#### 3.3.4 Risk perception and attitudinal changes following the intervention

Students’ perceptions of radon risk were evaluated using Likert-scale items (39) assessing perceived hazard and level of concern. Responses were collected before (PRE) and after (POST) the educational intervention using a five-point Likert scale ranging from “Not at all” to “A lot” (Table 8). Percentages in Table 8 were calculated based on the valid responses available for each individual item.

**Table 8:** Percentage distribution of Likert-scale responses for perceived dangerousness and concern regarding radon before (PRE) and after (POST) the intervention.

Likert score	PRE (%) (*n* = 316)	POST (%) (*n* = 307)	PRE (%) (*n* = 294)	POST (%) (*n* = 295)
	Dangerousness		Level of Concern	
1 (Not at all)	4.43%	6.19%	11.22%	9.83%
2 (Very little)	6.96%	6.19%	10.54%	16.61%
3 (A little)	20.57%	17.92%	26.19%	30.51%
4 (Quite)	47.47%	48.53%	27.55%	23.73%
5 (A lot)	20.57%	21.17%	24.49%	19.32%

The comparable Likert-scale items showed different response patterns across the assessed dimensions. Self-perceived knowledge increased markedly after the intervention, with the median response increasing from 1 in the PRE survey to 3 in the POST survey (*p* < 0.001). In contrast, perceived dangerousness remained unchanged (median 4 in both groups; *p* = 0.804), while the small difference observed in health-related concern (median 4 vs. 3) did not reach statistical significance (*p* = 0.054). Detailed Mann–Whitney U statistics and effect-size estimates are reported in Supplementary Table S4.

#### 3.3.5 Student satisfaction and participant-level differences

Students’ overall satisfaction with the educational intervention was assessed using post-intervention Likert-scale responses, with the distribution of satisfaction levels shown in Supplementary Figure S5. The intervention was well received: 52% of participants reported being “very satisfied” and 34% “satisfied,” while only 14% expressed low satisfaction. These results indicate a high level of acceptance and perceived utility of the educational activity.

Satisfaction was also examined across age groups to assess consistency across educational stages. As shown in Supplementary Figure S6, high satisfaction levels were observed in all age categories, with only minor differences between groups. The proportion of students reporting low satisfaction remained limited across all age ranges.

Gender-based comparisons evaluated possible differences in response patterns across the assessed domains. As shown in Supplementary Figure S7, female students reported slightly higher scores than male students in knowledge accuracy, awareness, actions, and risk perception, with the largest difference observed in the awareness and actions domain.

## 4 Discussion

The RASCLET pilot study combined indoor radon monitoring with a school-based citizen-science and educational approach, providing information on both indoor exposure conditions and changes in radon-related knowledge and awareness. Indoor radon concentrations varied substantially among the participating schools, despite all school-level mean concentrations remaining below the European Union reference level of 300 Bq m^−3^. The lack of a consistent correspondence between CSN radon zoning, modeled radon flux, and measured indoor concentrations indicates that geological classification alone does not fully characterize indoor exposure conditions. Previous studies have similarly shown that building-specific characteristics, including floor level, basement characteristics, ventilation, and structural features, can strongly influence indoor radon variability (40, 41). The tendency toward higher concentrations in basement and ground-floor rooms observed in the present study is also consistent with previous school-based studies reporting higher radon concentrations at lower floor levels (7, 41).

The indoor radon concentrations observed in the RASCLET schools were comparatively moderate relative to those in previous European school surveys. The median concentration in the present study was 49 Bq m^−3^ (IQR: 24–77 Bq m^−3^), with school-level means ranging from 21 to 163 Bq m^−3^. In a recent survey of 198 Romanian educational institutions, Cucoş et al. (41) reported higher radon concentrations overall, with 24% of the educational buildings exceeding 300 Bq m^−3^. School-based measurements in Portugal have likewise shown that indoor radon concentrations can vary according to factors such as classroom floor level, building age, and occupancy patterns (7). Although direct numerical comparisons between studies should be interpreted cautiously due to of differences in geographical setting, building and ventilation characteristics, sampling design, and measurement periods, these findings support the substantial site and building-specific variability observed among the RASCLET schools.

The environmental parameter analyses showed generally weak associations between indoor radon concentrations and the available meteorological variables and modeled radon flux. Wind speed was the only variable showing a statistically significant association in the multivariable analysis; however, this finding should be interpreted cautiously because the analyses were based on a limited number of independent observational units. Meteorological conditions can influence indoor radon through changes in ventilation, pressure differences and soil-gas transport, and relationships between indoor radon and meteorological parameters have been reported in previous studies (20, 42). In the present study, however, the available environmental variables alone were insufficient to explain the observed variability among schools. This suggests that building and site-specific characteristics not fully captured in the present dataset may also play an important role. Accordingly, the regression analyses should be regarded as exploratory rather than predictive.

An important outcome of the RASCLET project was the educational and citizen-science component. Following the intervention, substantial improvements were observed in students’ factual knowledge of radon and selected awareness outcomes. These findings are consistent with previous radon citizen-science research. Martell et al. (16) showed that citizen-science initiatives can contribute meaningfully to radon testing and research, while also noting that most existing radon initiatives involve relatively simple levels of participation and are generally led by universities or public authorities. Similarly, Stanifer et al. (17) reported significant improvements in environmental health literacy, health-information efficacy, and radon-testing self-efficacy following a citizen-science home radon-testing intervention. The present study extends this participatory approach to the school environment, where students, under teacher guidance, researched radon and its associated health effects, jointly selected detector locations with their teachers, and participated in detector placement in small groups following the protocol provided by the research team. The RASCLET approach therefore connected professionally controlled radon measurements with school-community participation and environmental health education, while maintaining scientific measurement and data analysis within the research team.

The educational findings further suggest that schools can provide an effective setting for communicating environmental health information beyond the classroom. The reported communication between students and their families indicates that school-based activities may facilitate the transfer of radon-related information to the household level. Importantly, however, the intervention did not influence all dimensions of perception equally. The strongest changes occurred in factual knowledge and selected awareness indicators, whereas perceived dangerousness and health-related concern did not show statistically significant PRE–POST differences. In the context of this intervention, these findings suggest that changes in factual understanding and specific awareness were more evident than changes in broader perceptions of risk or concern. The high satisfaction reported across age groups nevertheless indicates that the educational activities were well received by participating students.

The findings should, nevertheless, be interpreted in the context of the pilot nature of the study. At the same time, this first pilot experience provides valuable information for identifying methodological and organizational needs and for informing the design of a larger study involving schools distributed across Spain. First, the relatively small number of participating schools (*n* = 13) limits the statistical power of the school-level environmental analyses, particularly for multivariable regression. Multiple detectors were deployed within each school and therefore represented clustered measurements rather than fully independent observations; accordingly, analyses involving school-level environmental predictors were conducted using the school as the independent unit. Second, indoor radon concentrations were integrated over approximately 90 days, preventing assessment of short-term temporal relationships between meteorological variations and indoor radon concentrations and precluding direct interpretation of the measurements as annual mean concentrations. Third, some environmental predictors were obtained from external spatial datasets rather than measured directly at each building. In particular, modeled radon flux was available at a spatial resolution of 0.05° × 0.05°, which may not capture fine-scale site or building-specific variation. Finally, although average daily occupancy and qualitative ventilation information were recorded as contextual metadata, detailed time-resolved occupancy patterns, quantitative air-exchange rates, and comprehensive building characteristics were not available. The PRE and POST questionnaires were also anonymous and were therefore analyzed as independent groups rather than paired individual responses. Nevertheless, the educational component involved a substantial number of students, with 538 PRE and 339 POST questionnaire responses, providing a broad dataset for assessing changes in knowledge and awareness at the group level. These limitations should be considered when interpreting the exploratory environmental associations and the PRE–POST educational comparisons, while the descriptive radon measurements remain directly informative for the participating schools.

## 5 Conclusion

The RASCLET pilot study demonstrates the feasibility of integrating school-based indoor radon monitoring with citizen-science and educational activities in Catalonia. Of the 52 indoor detectors deployed, 47 provided valid measurements. Indoor radon concentrations showed a median of 49 Bq m^−3^ (IQR: 24–77 Bq m^−3^), while school-level mean concentrations ranged from 21 to 163 Bq m^−3^. All school-level mean concentrations remained below the European Union reference level of 300 Bq m^−3^, although two schools exceeded the WHO reference level of 100 Bq m^−3^. Higher concentrations were generally observed in basement and ground-floor rooms.

Exploratory school-level analyses indicated generally weak associations between indoor radon concentrations and the available environmental variables. Wind speed was the only variable showing a statistically significant association in the multivariable analysis (*β* = −102.022, *p* = 0.025); however, this finding should be interpreted cautiously given the limited number of independent school-level observations.

The educational component involved 538 PRE and 339 POST questionnaire responses and was associated with substantial improvements in students’ radon knowledge and selected awareness indicators.

Correct identification of radon as a radioactive gas increased from 34.2 to 72.9%, while correct responses concerning its long-term health effects increased from 42.0 to 85.5%. Awareness of radon-associated diseases increased from 8.4 to 31.3%, and increased communication with families was also observed following the intervention.

Overall, the findings show that combining direct indoor radon measurements with citizen-science and educational activities can provide both exposure information and a practical framework for environmental health education and radon risk communication in schools.

## Data Availability

The original contributions presented in the study are included in the article/Supplementary material, further inquiries can be directed to the corresponding author.
