# Supplementary material for: Indoor radon monitoring through citizen science in Catalan schools: environmental assessment and educational outcomes
Source: Front Public Health. 2026 Oct 26;14:1953882. doi: 10.3389/fpubh.2026.1953882 (PMC13752855; doi:10.3389/fpubh.2026.1953882)
Supplement: Supplementary file 1 [file Data_Sheet_1.DOCX]

**Supplementary Material**

**Indoor Radon Monitoring through Citizen Science in Catalan Schools: Environmental Assessment and Educational Outcomes**

Misbah Javed, Ian Martínez Argumosa, Adrina Jimenez, Roser Sala Escarrabill, Arturo Vargas, Ferran Campillo i López, David Pino, Carlo Sabbarese, Claudia Grossi

**Supplementary Figures**

**Supplementary Figure S1**. Radon potential (P90) map for Catalonia obtained from the SIU system. Red circles indicate the schools’ locations. The study area extends from 40.52°N to 42.86°N in latitude and from 0.15°E to 3.33°E in longitude. Data sources available at <https://mapas.fomento.gob.es/VisorSIU/>

**Survey Domain**

**Demographics**

**Subjective Knowledge of Radon**

**Objective Knowledge of Radon**

**Risk Perception**

**Protective Behaviors**

Basic personal and school information

(Age, gender school name)

**Measurement Behavior (*Pre only*)**

Self-perceived understanding

(How much do you think you know about radon?)

**Satisfaction & Expectations**

**(*Post only*)**

Actions to reduce exposure

(Is there anything you can do to protect yourself?)

Experience with home radon testing at home

(Has anyone in your family ever measured radon?)

Experience and usefulness of project

(How satisfied are you with participating?)

True/false factual statements

(Radon is a radioactive gas)

Perceived health risk & concern

(How dangerous do you think radon is for your health?)

**Content Type**

**Response Format**

**Multiple choice/ short answer**

**Yes/No + “If yes, how?”**

**Likert scale + open field**

**True / False / I don’t know**

**5-point Likert scale + optional open field**

**Yes/No + optional description**

**5-point Likert scale	(None → A lot)**

**Supplementary Figure S2.** Structure of the pre- and post-intervention student questionnaires

**Supplementary Figure S3.** Frequency distribution of measured indoor radon concentrations across all schools.

**Supplementary Figure S4.** Combined time series of meteorological parameters recorded at the YA station between 2018 and 2025: (a) air temperature, (b) relative humidity, (c) atmospheric pressure, and (d) wind speed. Colored background bands indicate the seasonal periods (winter, spring, summer, and autumn).

**Supplementary Figure S5.** Overall student satisfaction with educational intervention based on post-intervention questionnaire responses.

**Supplementary Figure S6*.*** Post-intervention student satisfaction by age group, showing the percentage of students reporting satisfied and not satisfied responses within each age category.

**Supplementary Figure S7.** Gender-based comparison of response distribution (%) across knowledge accuracy, awareness & actions, and risk perception domains.

**Supplementary Tables**

| **Activity** | **Nov** | **Dec** | **Jan** | **Feb** | **March** | **April** | **May** | **June** |
| --- | --- | --- | --- | --- | --- | --- | --- | --- |
| **PEH referents meeting** |  |  |  |  |  |  |  |  |
| **School referents meeting** |  |  |  |  |  |  |  |  |
| **Detectors shipment and protocol distribution** |  |  |  |  |  |  |  |  |
| **Pre-surveys** |  |  |  |  |  |  |  |  |
| **Radon measurements** |  |  |  |  |  |  |  |  |
| **Post-surveys** |  |  |  |  |  |  |  |  |
| **Detectors return** |  |  |  |  |  |  |  |  |
| **Data analysis** |  |  |  |  |  |  |  |  |
| **UPC dissemination event** |  |  |  |  |  |  |  |  |

**Supplementary Table S1**. Timeline of the RASCLET study activities across the 2024–2025 academic year.

***Abbreviations:*** *PEH = Pediatric Environmental Health; UPC = Universitat Politècnica de Catalunya.*

**Supplementary Table S2.** Summary of deployment metadata documented for each Radtrak³ detector.

| **Category** | **Metadata Fields** | **Details Recorded** |
| --- | --- | --- |
| **Detector information** | Detector_ID | Unique Radtrak³ code assigned by Radonova |
|  | Start date/End date | Measurement starts and end timestamps |
|  | Detector type | Classroom detector / Background detector (5) |
|  | School name | The center where the detector was placed |
| **Location details** | Municipality | Catalonia municipality |
|  | Latitude / Longitude | GPS coordinates of the room location |
|  | Floor level | Basement, Ground, First floor, Second floor, Third floor |
| **Room and occupancy characteristics** | Room name/number | Local room label (Classroom 4A, Library) |
|  | Reported ventilation conditions | Qualitative assessment based on window and door opening practices |
|  | Occupancy (hours/day) | Average daily use of the room by students/staff |

**Supplementary Table S3.** Variance Inflation Factors (VIF) for predictors included in the full multiple linear regression model.

| **Predictor** | **VIF** |
| --- | --- |
| Humidity relative | 18.9 |
| Pressure | 214.3 |
| Temperature | 135.03 |
| Wind speed | 3.21 |
| Radon flux | 2.61 |

**Supplementary Table S4.** Item-level statistical comparison of comparable PRE and POST questionnaire responses following the educational intervention.

| **Domain** | **Item** | **PRE result** | **POST result** | **Test statistic** | **p-value** | **Effect size** |
| --- | --- | --- | --- | --- | --- | --- |
| Factual knowledge | Q1 | 34.2% | 72.9% | χ² = 124.37 | <0.001 | V = 0.377 |
|  | Q2 | 42.0% | 85.5% | χ² = 162.77 | <0.001 | V = 0.431 |
|  | Q3 | 14.9% | 26.0% | χ² = 16.51 | <0.001 | V = 0.137 |
|  | Q4 | 20.3% | 30.7% | χ² = 12.28 | <0.001 | V = 0.118 |
|  | Q5 | 22.3% | 58.1% | χ² = 115.53 | <0.001 | V = 0.363 |
|  | Q6 | 12.3% | 18.9% | χ² = 7.20 | 0.007 | V = 0.091 |
|  | Q7 | 11.0% | 20.1% | χ² = 13.88 | <0.001 | V = 0.126 |
|  | Q1 | 8.4% | 31.3% | χ² = 76.54 | <0.001 | V = 0.295 |
| Awareness | Q2 | 20.1% | 28.0% | χ² = 7.39 | 0.007 | V = 0.092 |
|  | Q1 | Median = 1 | Median = 3 | U = 29,077.5 | <0.001 | r_rb = 0.677 |
| Self-perceived knowledge | Q2 | Median = 4 | Median = 4 | U = 47,985.0 | 0.804 | r_rb = 0.011 |
| Perceived dangerousness | Q3 | Median = 4 | Median = 3 | U = 47,239.0 | 0.054 | r_rb = −0.089 |
| Health concern |  |  |  |  |  |  |
